# Conductive Adhesive and Antibacterial Zwitterionic Hydrogel Dressing for Therapy of Full-Thickness Skin Wounds

**DOI:** 10.3389/fbioe.2022.833887

**Published:** 2022-02-24

**Authors:** Feng Wang, Shuguang Wang, Liping Nan, Jiawei Lu, Ziqi Zhu, Jintao Yang, Dong Zhang, Junjian Liu, Xiao Zhao, Desheng Wu

**Affiliations:** ^1^ Department of Spine Surgery, Shanghai East Hospital, School of Medicine, Tongji University, Shanghai, China; ^2^ Department of Orthopedic, Shanghai East Hospital, School of Medicine, Tongji University, Shanghai, China; ^3^ Department of Orthopedic, Shanghai Tenth People’s Hospital, School of Medicine, Tongji University, Shanghai, China; ^4^ College of Materials Science and Engineering, Zhejiang University of Technology, Hangzhou, China; ^5^ Department of Chemical, Biomolecular, and Corrosion Engineering, College of Engineering and Polymer Science, The University of Akron, Akron, OH, United States; ^6^ Department of Anesthesiology, Shanghai General Hospital Affiliated to Shanghai Jiaotong University, Shanghai, China

**Keywords:** conductive hydrogel, adhesive hydrogel, antibacterial hydrogel, wound dressing, wound healing

## Abstract

Any sort of wound injury leads to the destruction of skin integrity and wound formation, causing millions of deaths every year and accounting for 10% of death rate insight into various diseases. The ideal biological wound dressings are expected to possess extraordinary mechanical characterization, cytocompatibility, adhesive properties, antibacterial properties, and conductivity of endogenous electric current to enhance the wound healing process. Recent studies have demonstrated that biomedical hydrogels can be used as typical wound dressings to accelerate the whole healing process due to them having a similar composition structure to skin, but they are also limited by ideal biocompatibility and stable mechanical properties. To extend the number of practical candidates in the field of wound healing, we designed a new structural zwitterion poly[3-(dimethyl(4-vinylbenzyl) ammonium) propyl sulfonate] (SVBA) into a poly-acrylamide network, with remarkable mechanical properties, stable rheological property, effective antibacterial properties, strong adsorption, high penetrability, and good electroactive properties. Both *in vivo* and *in vitro* evidence indicates biocompatibility, and strong healing efficiency, indicating that poly (AAm-*co*-SVBA) (PAS) hydrogels as new wound healing candidates with biomedical applications.

## Introduction

Although the skin is the softest, most flexible outer tissue covering the body of a vertebrate animal, the inevitable skin trauma caused by burns, contusions, or bruises is a significant yet intractable healthcare issue around the world (Hernández Martínez et al., 2019). A wound visible with the naked eye easily tends to turn into a perfect breeding ground for pathogenic bacteria (*S. aureus*, *E. coli*) and fungus, which further impairs vital functions that the skin performs and causes pain, weak immune responses, disability, or even death. More importantly, the presence of a number of complex driven factors such as 1) the increasing prevalence of chronic conditions resulting in acute, chronic, surgical, and traumatic wounds, 2) the growing costs on chronic wounds, and 3) climbing use of regenerative medicine in wound disposal, leading to the global wound care market projected to reach over USD 27 billion by 2026 from USD 19.3 billion in 2021. Based on this status, the high growth potential of emerging economies and rapidly increasing wound treatment requirements have also opened a myriad of opportunities for exploiting more fundamental wound healing mechanisms and novel materials.

From the perspective of epidermal healing mechanisms, practical healing efficiency tends to be distinctly promoted in wounds subjected to a moist environment under an occlusive dressing film as compared to uncovered wounds. The sustaining moist environment will decrease the inflammatory and proliferative cells, resulting in faster healing of wound defects. To date, the widely accepted characteristics for ideal biological wound dressings can be summarized as 1) a certain adsorption capacity for removing excess exudates and toxins, 2) good air permeability to allow gaseous exchange and thermal insulation, and 3) adjusted adhesion to promote the wound/dressing interface binding force and to be removed easily without trauma to the wound (Zhu et al., 2016; Ni et al., 2021; Wang et al., 2021; Yuan J et al., 2021). Recent progress has demonstrated that biomedical hydrogels can be used as typical wound dressings to accelerate the whole healing process due to the similarities, in terms of composition structure (soft matrix with high water contents), to human tissues. In addition, hydrogels are hydrophilic, fully crosslinked polymeric materials that have enabled us to dynamically alter adhesion ability, leading to stable adhesion to the active epidermis. For instance, most as-prepared soft natural (gelatin, hyaluronic acid, and agar) or synthetic (polyAAm, polyHEAA, and polyNIPAm) hydrogels possess strong adhesion on common biomedical substrates (e.g., steel, titanium, glass, and ceramic), while the bonding energy is dramatically reduced once the excess exudates and toxins are absorbed (Zhang et al., 2020a; Zhang et al., 2020b; Zhang et al., 2020c; Mao et al., 2020; Zhang et al., 2021).

Skin is electro-sensitive tissue in our body (Kim et al., 2013). Some researchers have been exploring electrical stimulation and its effect on wound healing (Li M et al., 2019; Chen et al., 2021; Yuan J et al., 2021; Zheng et al., 2021). In addition, it has been confirmed that endogenous electric stimulus has a positive impact on wound healing (Qu et al., 2019). This electrical stimulation can mainly promote the proliferation of fibroblasts, the synthesis of ECM, and the secretion of growth factors and revascularization (Goding et al., 2017) by the activation of transmembrane channels and enhancement of intracellular Ca2+concentration (Li et al., 2019c; Naskar et al., 2020). Therefore, electrical stimulation has the potential to treat full-thickness skin wounds with simplicity due to its relatively easy application.

Although natural polysaccharide- and protein-based hydrogels are biodegradable, the undesirable weak mechanical properties, especially and surprisingly, brittleness, still limit further practical applications. How to balance ideal biocompatibility and stable mechanical properties is still being explored by researchers in biophysical, material, or biomedical science. Some synthetic acrylamide polymers can address the challenge of matrix-strength, but non-specific protein adhesion and, subsequently, potential organ rejection make it difficult to peel the hydrogels from wounds in escharosis regions after healing. With the development of zwitterionic hydrogels, e.g., polyCBMA, polySBMA, and poly(trimethylamine N-oxide) (polyTMAO) (Huang et al., 2021), the macrophase cells upon non-fouling polyzwitterionic matrix tend to differentiate the pro-healing states. Thus, it is also possible to achieve a good combination of the above-mentioned properties to achieve ideal wound healing by using synthetic hydrogels.

In this work, we ingeniously introduce a new structural salt-responsive zwitterion poly[3-(dimethyl(4-vinylbenzyl) ammonium) propyl sulfonate] (SVBA) into the polyAAm network to extend the practical candidates for the field of wound healing. The nature of polySVBA chains previously already presented unique salt-responsive properties and was applied to bacterial release (Zhang et al., 2018; Wu et al., 2019), selective oil/water separation (Wang et al., 2020), and thermal-induced soft actuators (He et al., 2019), etc. Interestingly, rigid aromatic backbone and sequential ionic interactions synergistically promote surface bonding, potential conductivity, matrix stretchability, and self-healing properties. On the one hand, compared to commercial Tegaderm (3M), more wettability groups (hydroxy, sulfonate, and amide) from copolymerized poly (AAm-*co*-SVBA) assist in trapping more moisture and maintaining long-term hydration micro-environments. On the other hand, this structural adhesive design can make the zwitterionic hydrogel matrix fit the organ wounds smoothly and perfectly, leading to the overall adsorption of excess exudates and toxins during dynamic healing processes. In addition, the challenge of inevitable protein adhesion (e.g., cells and pathogenic bacteria) will finally lead to the failure of healing, as the formed scab grows against the surface of the hydrogel and the healed area tends to tear as the hydrogel is removed. However, polyzwitterionic hydrogels are widely reported to be antifouling materials (Yuan H et al., 2021) that promote protein repellent, and thus a higher healing efficacy can be achieved. Both *in vivo* and *in vitro* evidence monitors the cytotoxicity, biocompatibility, and healing efficiency of the resultant polyzwitterions with refined structures, indicating that poly (AAm-*co*-SVBA) (PAS) hydrogels have become new wound healing candidates for biomedical applications.

## Experimental Section

### Materials

Acrylamide (AAm, 99.0%) and N,N′-Methylenebisacrylamide (MBAA, 97%) were purchased from Aladdin reagent Inc. (Shanghai), The detailed synthesis route of dimethyl-(4-vinylphenyl) ammonium propane sulfonate (SVBA) can be found in our previous report (He et al., 2019; Yuan H et al., 2021). In this experiment, all purified water was obtained from a Millipore system with an electronic conductance of 18.2 MΩ cm.

### Preparation of Hydrogel

To obtain the functional polyzwitterionic hydrogel, a predetermined amount of cross-linking agent (MBAA, 1.0 mg ml^−1^), monomer (0.168 g/ml), and 2-hydroxy-4′-(2-hydroxyethoxy)-2-methylpropiophenone (I2959, photoinitiator, 10 mg ml^−1^) were prepped. The solution was purged with nitrogen gas for 10 min to completely remove the oxygen gas, after that, the solution was slowly injected into a prepared mold by separating two glass slides with a 1.0 mm Teflon spacer. The dimension of the mold was 50 × 50 × 1 mm^3^ (length × width × depth). After the system was irradiated by a 365 nm UV light for 1 h, the resultant hydrogel was released from the mold and immersed in pure water to remove unreacted monomers and free polymers. The cylinder hydrogel was prepared by using an injection syringe (1 ml) as the mold for the mechanical test.

### Characterization

A series of samples with different ratios of AAm and SVBA were prepared using the same method (PAS-1, AAm: SVBA = 1:1; PAS-2, AAm: SVBA = 2:1). The hydrogel samples were lyophilized under vacuum for 48 h to remove water for morphology measurements. Prior to imaging, the lyophilized hydrogels were brittle-fractured in liquid nitrogen, then, the hydrogels were coated with Au and observed by scanning electron microscope (SEM, HITACHI S-4800, Japan) at an accelerating voltage of 5kV. The Fourier transformed infrared (FTIR) spectra were detected on a BRUKER Vector 22 Spectrometer (Germany) from 4,000 to 400 cm^−1^ in an ATR model. The tensile tests and compression tests were recorded on a universal testing machine (SANS CMT2503) at 75% humidity and room temperature. For tensile testing, the hydrogels of the cylindrical samples with a diameter of 4.5 mm and a height of 50 mm were stretched at a rate of 100 mm min^−1^. For compression testing, the cylindrical samples with a diameter of 8.5 mm and a height of 40 mm were compressed at a velocity of 10 mm min^−1^.

A rotational rheometer (Thermo Hakke) with a steel parallel plate of 10 mm radius was used to determine the rheological properties of the hydrogel. Dynamic frequency sweep measurements were performed at 37°C by an oscillation mode using a fixed oscillatory strain of 1% and a fixed gap of 1 mm. The temperature sweep measurements of the hydrogel were measured with a temperature range of 10–60°C, a fixed oscillatory strain from 0.1% to 600%, and a fixed frequency of 1 Hz. The strain measurements of the hydrogel from at the frequency of 1 Hz (37°C) were performed.

### Adhesive Strength Test

The *in vitro* adhesion of the PAS-2 hydrogel was further assessed through different rat tissues. Four organs (i.e., heart, liver, lung, kidney) were excised from SD rats for tissue adhesive tests. A square-shaped PAS-2 hydrogel (of 6 mm per side) was attached to stainless steel tweezers before being attached to the water-moist surface of these organs.

### *In vitro* Cytotoxicity Assay

The biocompatibility of hydrogels was evaluated in the fibroblast cell line (L929). In brief, cells were seeded onto 24-well culture plates and cultured overnight. Then, after ultraviolet treatment, PAS-2 hydrogel was added into the 24-well plates and co-cultured. The standard medium was changed daily. After 1, 3, and 5 days, the media and hydrogels were discarded. At the specified time points, a CCK-8 working solution (DMEM containing 10% CCK-8 solution) was added and incubated in the incubator for 2 h. The supernatant was collected and measured for the absorption value at 450 nm using a microplate reader (Bio-Rad, USA). A live/dead assay was carried out to evaluate the cytotoxicity of prepared hydrogels. Briefly, the cells were stained with calcein-AM/Propidium dye at the same time points as described above and observed under a confocal microscope (Leica, Germany). Every test was repeated three times.

### Hemolytic Activity

Rat blood cells were used to evaluate the blood compatibility of PAS-2 hydrogel. Briefly, the erythrocytes of SD rats were collected by centrifugation (1,000 rpm, 10 min) from the whole blood, and then diluted by normal saline (NS). The prepared erythrocytes solution was incubated with PAS-2 hydrogel. In addition, erythrocytes treated with PBS served as a negative control and those treated with Triton X-100 (0.1%) served as a positive control After incubating at 37°C for 2 h, the supernatant was taken out via centrifugation and transferred to a 96-well plate to detect the absorbance at 570 nm. Every test was repeated three times.

$$\mathrm{Hemolysis}\;\mathrm{ratio}\;\left(\%\right)=\frac{ODa-ODb}{ODc-ODb}*100\%$$

Where ODa is the absorbance of samples, and ODb and ODc represent the absorbance of PBS and Triton X-100.

### Antibacterial Performance

The *in-vitro* antibacterial performance of PAS-2 hydrogel was tested against *S. aureus* (ATCC 29213) and *E. coli* (ATCC 25922) in this experiment by standard plate counting assays. *E. coli* and *S. aureus* were cultured in Luria-Bertani (LB) and Tryptic Soy Broth (TSB) medium, respectively. Briefly, a total volume of 300 μL hydrogel solutions and equal volumes of PBS were loaded into a 24-well plate and then irradiated by UV for 30 min to kill any microorganisms. Then, the bacterial suspension (300 μl, 10^6^ CFU/ml) was mixed with PAS-2 hydrogel and PBS, respectively, which was incubated in a 37°C incubator for 12 h. The group of bacteria incubated with PBS was set as a control. Thereafter, the bacterial solution of two groups was isolated, then 20 μl was taken and seeded on the LB and TSB solid plate and placed at 37°C overnight. Finally, the number of viable colony units were photographed and counted.

### Wound Healing Study

All experiments were authorized by the Animal Ethics Committee of Shanghai East Hospital (Shanghai, China). A total number of 27 adult Sprague-Dawley (SD, male, weigh, 250–300 g, age, 6 weeks old) rats, were purchased by Shanghai Slac Laboratory Animal Co. Ltd. Animal care and use followed the guidelines of Laboratory Animals published by the US National Institutes of Health.

Animals were bred in a rat box in a controlled environment (temperature: 26 ± 3°C, relative humidity: 70–85%, 12: 12 h light/dark). Experimental animals were not restricted in how much water they could have and had a standard diet. Briefly, after a standard anesthesia procedure (60 mg kg^−1^ pentobarbital), the fur was shaved from the backs. A local full-thickness wound with a diameter of approximately 1.5 cm was produced on the dorsum of each rat. The rats were randomly assigned to three groups: the control group (gauze), the Tegaderm group (commercial film), and the PAS-2 group (PAS-2 hydrogel). At least nine rats were selected for each group. The dressing was refreshed on the 4, 8, and 12th days and the healing of the wound was recorded with a digital camera on the 0, 4, 8, and 12th days and the corresponding wound closure rate was calculated.

$$\mathrm{Wound}\;\mathrm{closure}\;\mathrm{rate=}\frac{Area\;0-Area\;T}{Area\;0}*100\%.\;$$

Here, the Area 0 is the original wound area, the Area T represents the wound area at each time point (Cheng et al., 2020).

### Histological Analysis

To evaluate the wound healing performance of PAS-2 hydrogel, the tissue samples of the wound were collected on the 4, 8, and 12th day and fixed with 4% paraformaldehyde for 1 h. Subsequently, the samples were embedded in paraffin, sectioned (4 μm), H&E and Masson stained, followed by histologic examination with optical microscopy (Leica Microsystems, Germany). A H&E-staining-based scoring system was used to evaluate wound repair (Eikebrokk et al., 2019). Masson staining was performed to evaluate collagen deposition using ImageJ software. In addition, the collagen amount and neovascularization in the wound tissue at day 12 were visualized by immunofluorescence staining of collagen type I (Col I) and alpha-smooth muscle actin (α-SMA). In short, after routinely de-waxing and rehydration, antigens were repaired and blocked with goat serum. The slides were incubated, respectively, overnight with primary antibodies at 4°C: rabbit polyclonal anti-collagen type I and anti-α-SMA (1: 100, CST, USA). After undergoing PBS washes, tissues were incubated with Cy3-conjugated goat anti-rabbit IgG (1: 200, Abcam, USA). Nuclei were stained with DAPI (Abcam, USA) after the incubation of the secondary antibody. The immunostaining results were observed by a fluorescence microscope (Nikon, Japan) and quantitatively analyzed by ImageJ software. The results were evaluated independently by two pathologists who were blinded to the treatments.

### Statistical Analysis

The analysis data are expressed as the mean ± standard deviation of at least triplicate samples. The student’s t-test was used to analyze the differences between two groups, and one-way analysis of variance (ANOVA) was used to analyze multiple groups. *p < 0.05 was considered to be statistically significant (*p < 0.05, **p < 0.01, ***p < 0.001 and ****p < 0.0001).

## Results and Discussion

### Synthesis and Characterization of the PAS-2 Hydrogel

**FIGURE 1:**
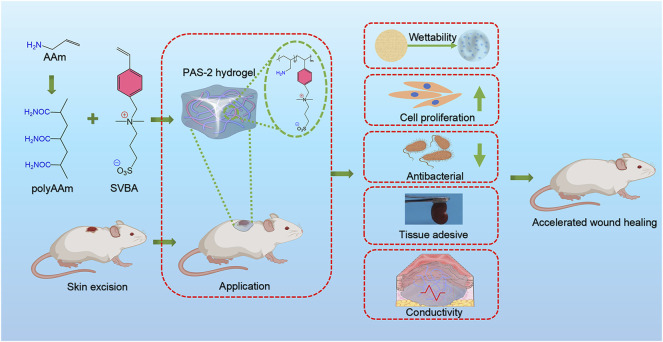
Schematic representations of PAS-2 hydrogel for wound healing.

**FIGURE 2:**
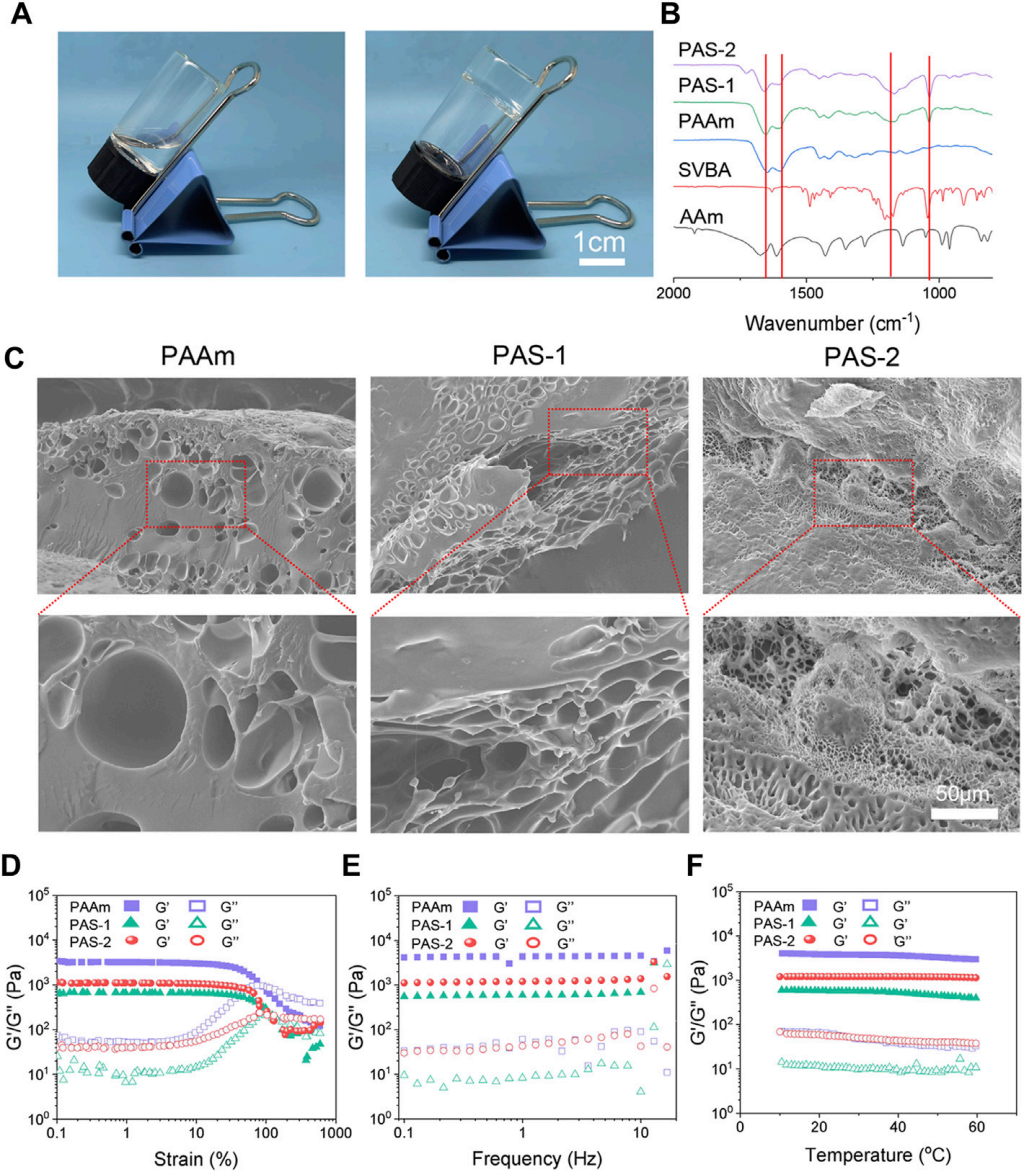
Microscopic morphology and mechanical characterization of prepared hydrogels. **(A)** Photographs of PAS-2 hydrogel. **(B)** FT-IR spectra of the AAm, PSBVA, PAAm hydrogel, PAS-1 hydrogel, and PAS-2 hydrogel. **(C)** SEM images of PAAm, PAS-1, and PAS-2 hydrogels. **(D)** G′ and G″ of the PAAm, PAS-1, and PAS-2 hydrogels on the strain sweep test. **(E)** Rheological analysis of PAAm, PAS-1, and PAS-2 hydrogels at 1% strain. **(F)** Rheological analysis of PAAm, PAS-1, and PAS-2 hydrogels when the temperature altered.

In this study, a new polyzwitterionic hydrogel was designed. The schematic representation of the hydrogel dressing is presented in Figure 1. A new structural salt-responsive polySVBA was induced into the polyAAm network to form a poly(AAm-*co*-SVBA) (PAS) hydrogel (Figure 2A). The chemical structures of the resultant hydrogels and polymer were tested using FT-IR spectroscopy. Figure 2B summarizes the FT-IR spectra of lyophilized AAm, PAAm, PSVBA, PAS-1, and PAS-2, respectively. For PAAm polymer, there were two characteristic peaks at 1,649 cm^−1^ and 1,599 cm^−1^ attributed to the asymmetrical stretching vibration of–C=O and the characteristic absorption peak of–N–H on amide groups. In addition, the characteristic absorption peaks at 1,174 cm^−1^ and 1,043 cm^−1^ for pure PSVBA hydrogel corresponded to the–CH stretching vibration on aromatic rings. However, once two networks were rationally combined, the ATR-FTIR spectra of polyzwitterionic PAS (including PAS-1 and PSA-2) possessed a certain shift—the obvious characteristic absorption peaks at 1,652 cm^−1^ and 1,601 cm^−1^ corresponding to the stretching vibration of–C=O and–N–H can be detected. In the meantime, the signal of the aromatic ring (-CH) shifted to 1,168 cm^−1^ and 1,038 cm^−1^, indicating the successful combination of PAAm and PSVBA networks in polyzwitterionic PAS hydrogels.

As shown in Figure 2C, the morphology of the hydrogels was observed by SEM. The PAAm exhibited a smooth surface with few porous structures. However, the introduction of polySVBA into the PAAm networks caused a remarkable increase in porosity. With a decrease in the weight ratio of the polySVBA from 66.7% to 50%, the PAS-1 and PAS-2 hydrogels showed interconnected porous structures. In addition, compared with the PAS-1 hydrogel, PAS-2 hydrogel revealed a highly porous structure and had a reduction in pore diameter, thereby leading to a decrease in the ability of the hydrogel.

### Rheological Properties of PAS-2 Hydrogel

For an eligible wound dressing, it must maintain the structural integrity under appropriate external force so that it can protect injured tissue. Therefore, favorable mechanical performances are very important for an ideal skin wound dressing. In this study, the viscoelasticity and stability of the dynamic crosslinked hydrogels were investigated by rheological tests. The strain amplitude sweep results were shown in Figure 2D. The loss modulus (G″) was lower than the storage modulus (G′) under small, applied stress, indicating that the solid-like hydrogel was successfully formed (Xing et al., 2016). Moreover, the storage modulus G′ of the hydrogels began to decrease gradually with the increasingly applied stress. For single-network hydrogels PAAm, when the strain increased to 90%, the G′ curves overlapped, and the network was collapsed. In contrast, the curves overlapped only when the strain exceeded 100% for the double-network hydrogel PAS-1 and PAS-2, which demonstrated that the double-network hydrogel was more stable and could bear larger stress than the single-network hydrogel. Similar results were observed in frequency sweep and temperature sweep. The G′ was always higher than G″ of the hydrogels, further indicating that the hydrogels had both a stable structure and elasticity (Figures 2E,F). Put simply, all the rheological test results demonstrated that the mechanical properties of the PAS-1 and PAS-2 hydrogels were more excellent than PAAm hydrogels. The increased cross-linking density of the hydrogels by the secondary photo-cross-linking might contribute to the better mechanical properties of the co-network hydrogels (Wang et al., 2018; Cheng et al., 2019).

### Compression and Tensile Properties

**FIGURE 3:**
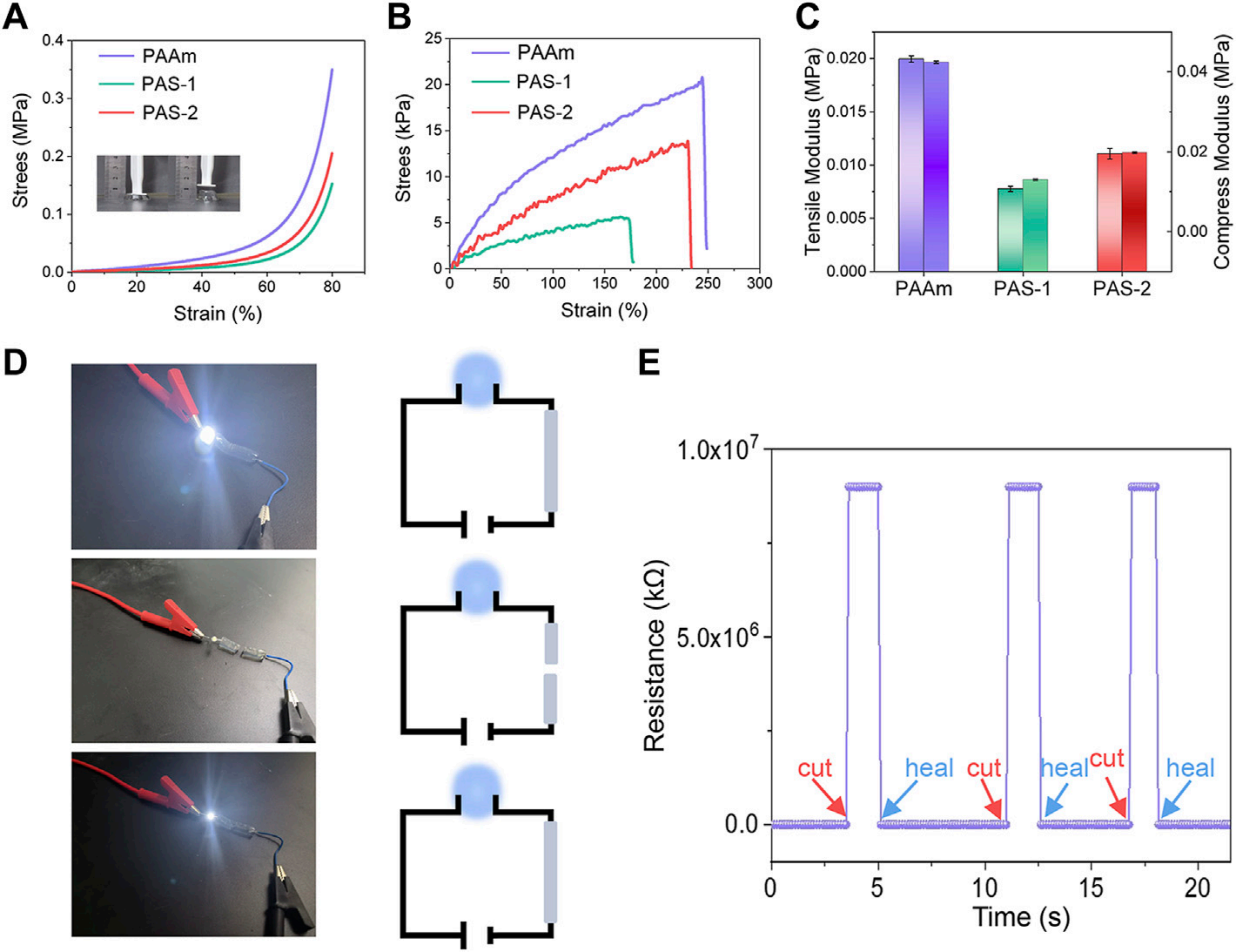
Mechanical properties and electrochemical sensitivity of prepared hydrogels. **(A)** Compress stress-strain curves of PAAm, PAS-1, and PAS-2 hydrogels. **(B)** Tensile stress-strain curves of PAAm, PAS-1, and PAS-2 hydrogels. **(C)** Tensile modulus and compress modulus of PAAm, PAS-1, and PAS-2 hydrogels. **(D)** Experiment of PAS-2 hydrogel in a DIODE circuit under different situations. **(E)** Relative resistance changes of PAS-2 hydrogel in response to different situations.

The compression and tensile properties of hydrogels are slightly influenced by the composition of the co-networks. The compression and tensile strength of the hydrogels were measured with a universal test machine. As shown in Figure 3A, the compression stress of PAAm was 0.062 MPa at a strain of 60%. After the salt-responsive SVBA was induced, the compression stress of PAS-1 and PAS-2 hydrogels decreased to 0.022 and 0.034 MPa with a strain of 60%. The tensile test was presented in Figure 3B. It was found that the tensile stress of PAAm hydrogel was 12 kPa at a strain of 100%, and the elongation at break was 250%. However, the tensile stress and elongation at the break point were both decreased as the SVBA was added. The tensile stress of both PAS-1 and PAS-2 hydrogels decreased from 8 to 4 kPa. Meanwhile, the elongation at break decreased from 240% to 180%. When compared with PAAm (0.04 MPa), the compression modulus of both PAS-1 and PAS-2 hydrogels decreased to 0.01 and 0.02 MPa (Figure 3C), which is comparable to the soft tissues of the human body (Feiner and Dvir, 2017). Typically, the ideal hydrogel dressing for wound healing is required to have low modulus, appropriate compressibility, and stretchability to avoid rupture during movement.

### Conductivity of the Hydrogels

It has been confirmed that electrically conductive polymers have a positive effect on cell adhesion, proliferation, and migration (Guo et al., 2011; Xie et al., 2015; Wu et al., 2017). Skin is an electrical signal sensitive tissue and previous studies have revealed that conductive wound dressings could favor wound healing (Li M et al., 2019; Qu et al., 2019; He et al., 2020). The conductivity of the PAS-2 hydrogel was evaluated by using a four-point probe method. As shown in Figures 3A,D LED bulb was lighted by a 1.5 V power supplier in a PAS-2 hydrogel connected circuit. The LED light became dimmed when the hydrogel was cut into two halves. Then, the two furcated parts were concatenated, and the LED bulb was lit again. Figure 3E illustrates that the resistance could be stably recovered once the two fractured hydrogels were put together. All the results indicated that the PAS-2 hydrogel possessed a good electroactivity. The electroactivity of the hydrogel may be attributed to SVBA. Zwitterionic hydrogels have an anti-polyelectrolyte effect, maintaining high solvent content and low polymer chain density even in a high salt environment, which provides an ion migration pathway and maintains very high ionic conductivity (Lee et al., 2018). The PAS-2 hydrogel contained cation and anion pairs, which can transmit electrical signals through the relative motion of positive and negative ions (Roshanbinfar et al., 2018).

### Adhesive Properties of the Hydrogels

**FIGURE 4:**
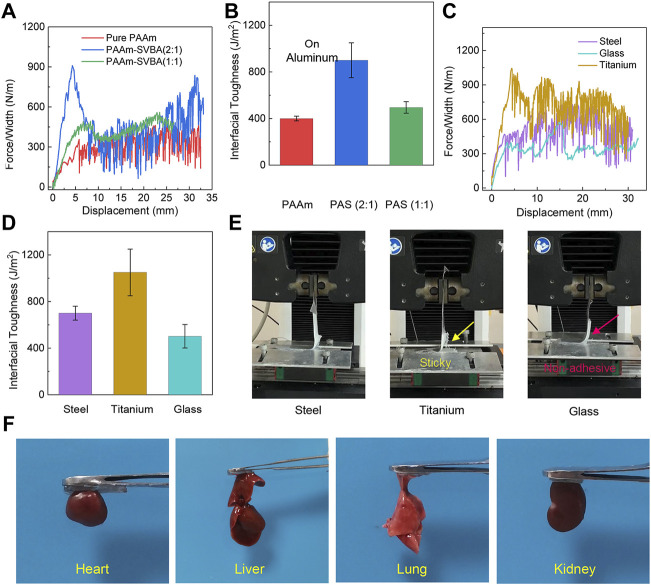
Interfacial toughness and adhesive properties of prepared hydrogels. **(A)** Peeling force/width curves of PAAm, PAS-1, and PAS-2 hydrogels on nonporous glass at a peeling rate of 100 mm/min. **(B)** Peeling Interfacial toughness of PAAm, PAS-1, and PAS-2 hydrogels on nonporous Aluminum at a peeling rate of 100 mm/min. **(C)** Peeling force/width curves of PAS-2 hydrogel on three nonporous solid surfaces at a peeling rate of 100 mm/min. **(D)** Interfacial toughness of PAS-2 hydrogel on different nonporous solid surfaces (steel, glass, titanium) at a peeling rate of 100 mm/min. **(E)** Visualization of peeling off of PAS-2 hydrogel from steel, glass, and titanium. **(F)** Photographs of tissue adhesion situations of PAS-2 hydrogel on four different organs of SD rat (heart, liver, lung, kidney).

Excellent adhesive property is important for a hydrogel wound dressing. Adhesive hydrogels can adhere to an irregular wound site and act as a physical barrier, protecting the wound from the adverse external environment (Le et al., 2018). We tested the adhesive properties of all hydrogels using the 90° peeling test. The force/width–displacement curves of different hydrogels and the interfacial toughness derived from force/width-displacement curves are illustrated in Figures 4A,B. It can be observed that the addition of SVBA changed the physical network structure and the network polymerization efficiency, the interfacial toughness of different hydrogels on aluminum increased from 400 ± 20 to 900 ± 150 J m^2^. To further examine the PAS-2 hydrogel possess good surface adhesion properties on different solid substrates. We performed a peeling test on the surfaces of titanium, glass, and steel. As shown in Figure 4C, the adhesion of PAS-2 hydrogel to titanium substrates was stronger than that on steel and glass. The interfacial toughness of PAS-2 hydrogel was ∼1050 J m^2^ on titanium, ∼700 J m^2^ on steel, and ∼502 J m^2^ on glass, respectively (Figure 4D). Visual inspection showed that the hydrogel can be easily peeled from the glass surface owing to the relatively smaller interfacial toughness (Figure 4E). In contrast, the PAS-2 hydrogel was sticky and adhered to the surface of the titanium. Different interfacial toughness of the same PAS-2 hydrogel on different substrate results from the hydrogel-substrate interaction. By incorporating SVBA into PAAm networks, the adhesive properties of hydrogels were improved. Besides mechanical properties, the ability for bio-adhesion to wound dressings is also important for potential clinical applications. the PAS-2 hydrogel also had high bio-adhesion properties to biological tissues (Figure 4F), such as the heart, liver, spleen, and kidney, which are crucial for clinical applications.

### Cytocompatibility and Hemocompatibility of PAS-2 Hydrogel

**FIGURE 5:**
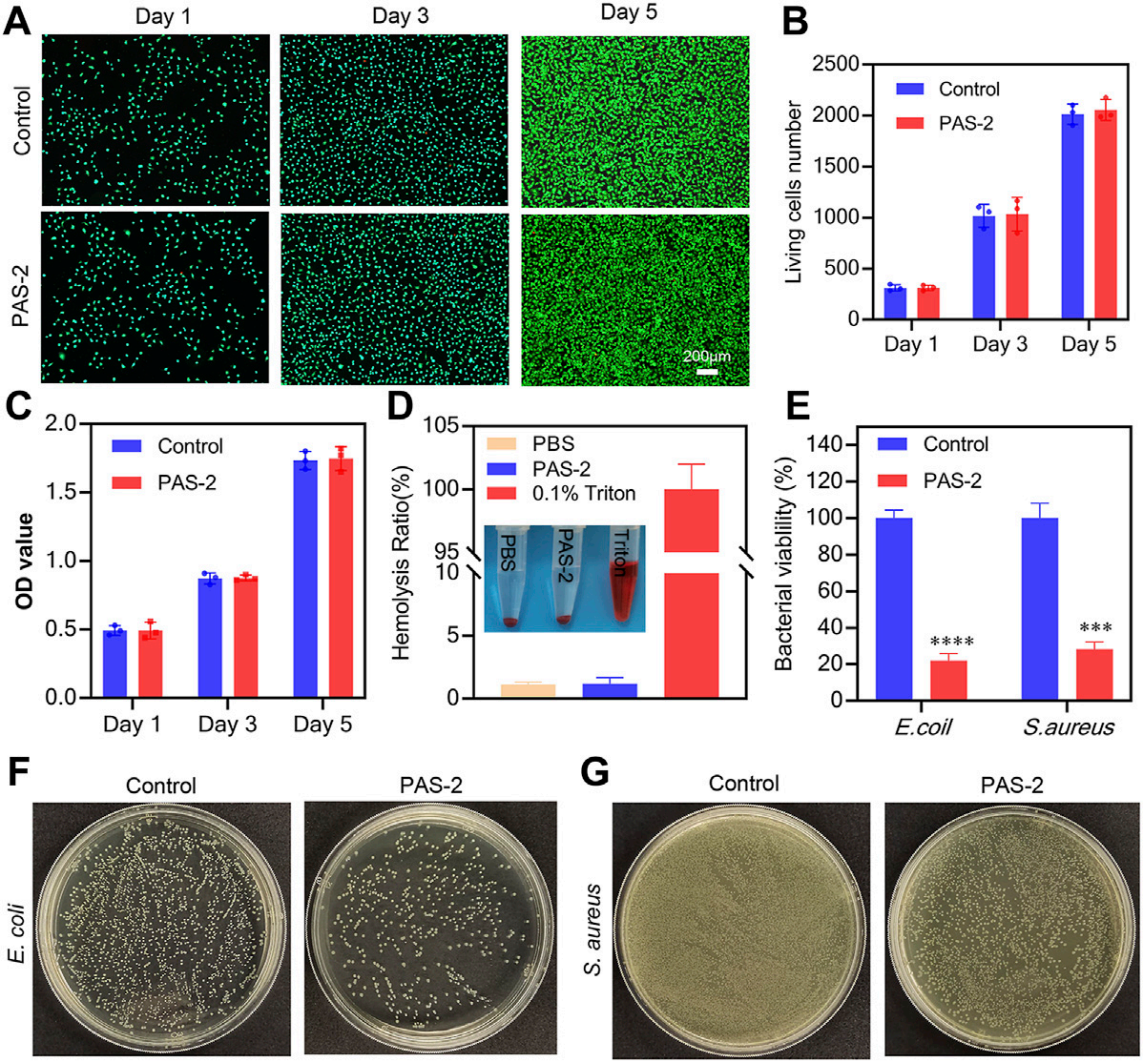
Cytocompatibility, hemocompatibility, and antibacterial properties of PAS-2 hydrogel. **(A)** Cytocompatibility of PAS-2 hydrogel indicated by live/dead staining after co-cultured with PAS-2 hydrogel on day 1, day 3, and day 5 and corresponding data analysis **(B)**, a normal culture medium without hydrogel was used as the control. **(C)** Quantitative analysis of L929 cell viability after co-cultured with PAS-2 hydrogel on day 1, day 3, and day 5, a normal culture medium without hydrogel was used as the control. **(D)** Image and the quantitative analysis of the hemolytic activity of the PAS-2 hydrogel. **(E)** The corresponding quantitative bacterial survival rate of *E. coli* and *S. aureus.* **(F, G)** Macroscopical image of the survival *E. coli* and *S. aureus* clones on agar plates after treated with PAS-2 hydrogel. *p < 0.05, **p < 0.01, ***p < 0.001, ****p < 0.0001.

Biomaterials possessing favorable biocompatibility are the prerequisite for new wound dressings (Zhao et al., 2018; Li S et al., 2019). Thus, the viability of L929 cells was employed to estimate the cytocompatibility of PAS-2 hydrogel with living/dead staining and CCK-8. As shown in Figure 5A, after co-incubation for 1, 3, and 5 days, dead cells (red) were scarcely detected in two groups and there was no difference in living cell number between the PAS-2 hydrogel and control groups (*p* > 0.05) (Figure 5B). The image of the CCK8 assay was also consistent with the results of the living/dead staining (Figure 5C).

To further explore the safety of PAS-2 hydrogel, the hemocompatibility of the hydrogel was evaluated 2 h after treatment by employing an *in vitro* hemolysis assay. The macroscopic color of PAS-2, PBS (negative control group), and Triton X-100 (positive control group) groups are shown in Figure 5D. The supernatant of the PAS-2 group was colorless and transparent, which was similar to the PBS group, while the Triton X-100 group was bright red. Furthermore, the hemolysis ratio was 1.14 and 1.21 for PBS and triton X-100 groups (*p* > 0.05), respectively, indicating that PAS-2 hydrogel had no significant effect on the hemolysis ratio. Overall, all these data confirmed that PAS-2 hydrogel had better cytocompatibility and they are taken as promising novel materials in wound dressing.

### Antibacterial Performance of PAS-2 Hydrogel

A promising wound dressing should have effective antibacterial activity to protect the wound from bacterial infection and inhibit the propagation of microorganisms to reduce inflammation in the site of the wound (Ak and Gülçin, 2008). In this work, we used *E. coli* and *S. aureus* to evaluate the antibacterial activity of PAS-2 hydrogel. polySVBA is an important component in the PAS-2 hydrogel, the nature of polySVBA chains already presented unique salt-responsive properties and were applied to bacterial release. As shown in Figures 5F,G, we found significantly fewer bacteria adhered to the interface of the PAS-2 hydrogel (*p* < 0.05) (Figure 5E), indicating that the PAS-2 hydrogel displayed an obvious inhibitory effect on *E. coli* and *S. aureus*. This antibacterial action is one of the most important properties of this wound dressing. The antibacterial rate of PAS-2 hydrogel was 78% (*S. aureus*) and 72% (*E. coli*), which was lower than that of previous studies (Khodabakhshi et al., 2019; Eskandarinia et al., 2020). This inconsistency is probably related to the addition of antimicrobial substances in previous works. The PAS-2 exhibited weaker antibacterial activity against *S. aureus* in comparison with *E. coli*. This may be derived from the structural difference of the two bacterial. The *S. aureus* is only covered by a single peptidoglycan layer, while *E. coli* has a thick membrane of lipopolysaccharide (Eskandarinia et al., 2019; Kefayat et al., 2021). Based on the above results, PAS-2 hydrogel can reduce bacterial growth to inhibit infection and shorten the wound healing time.

### *In vivo* Wound Healing

**FIGURE 6:**
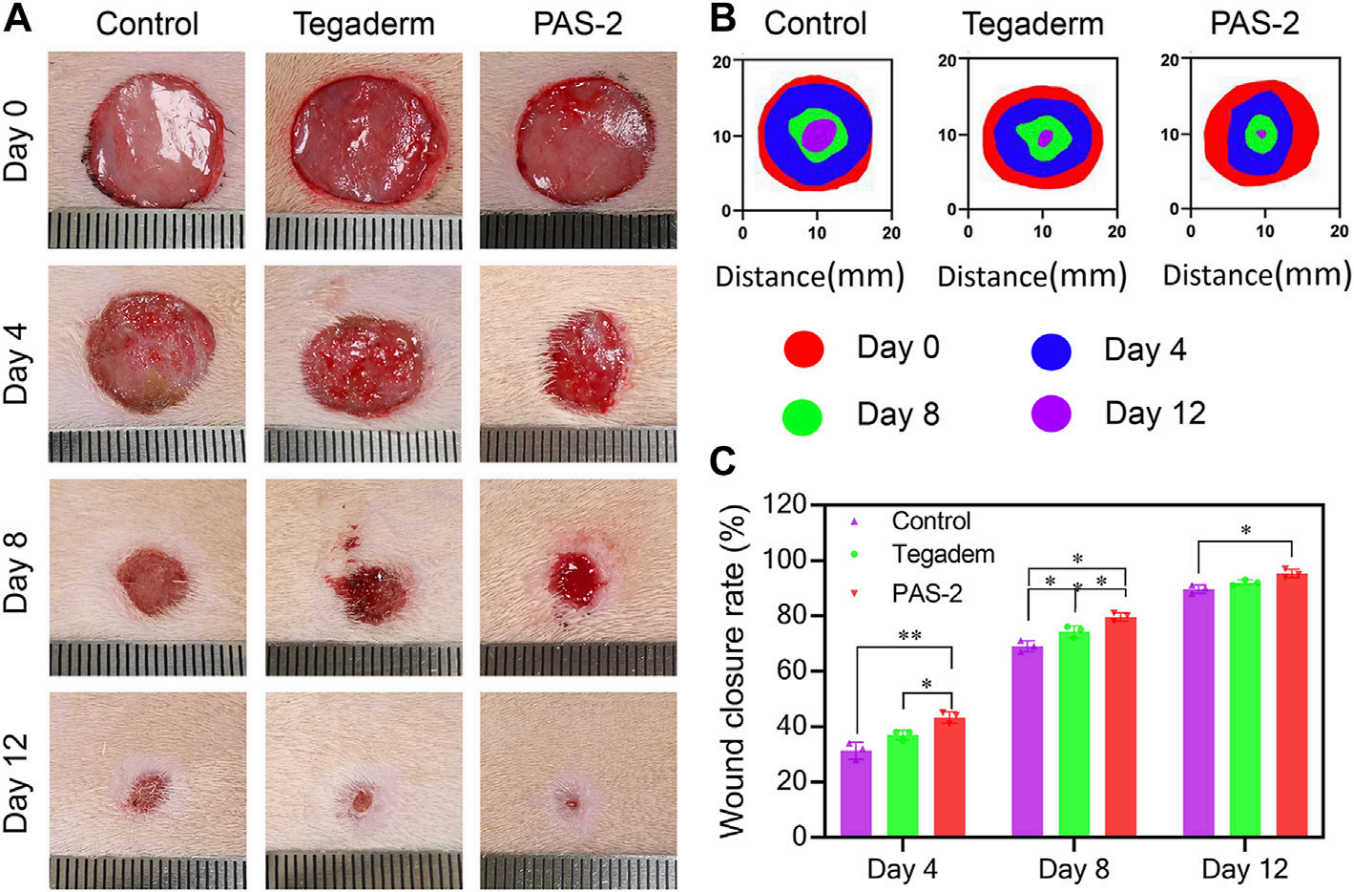
*In vivo* assessment of PAS-2 hydrogel for skin full-thickness wound healing. **(A)** Photographs of the wound on day 0, day 4, day 8, and day 12 for gauze (control), Tegaderm™ film, and PAS-2 hydrogel. **(B)** Corresponding traces of wound healing on day 0, day 4, day 8, and day 12. **(C)** The statistical analysis of wound closure for gauze (control), Tegaderm™ film, and PAS-2 hydrogel. *p < 0.05, **p < 0.01, ***p < 0.001, ****p < 0.0001.

PAS-2 hydrogel dressing could provide the wound with a long-term hydration micro-environment, antibacterial properties, and conduct bioelectro-stimulation. The wound healing behavior of the PAS-2 hydrogel was further evaluated in a rat full-thickness circular skin defect model. After 15 mm skin defects were created, the wounds were treated by the gauze (control), Tegaderm™ (commercial film dressing), and PAS-2 hydrogel and photographed on day 0, day 4, day 8, and day 12. As shown in Figures 6A–C, on the fourth day, although re-epithelialization of the wounds occurred in all groups, the hydrogel group (43.33%) showed a higher wound close rate than the Tegaderm group (37%) and control group (31.33%) (*p* < 0.05). On the eighth day, importantly, the wound closure rate of the PAS-2 hydrogel group exceeded nearly 11% (*p* < 0.05) than the control group. In addition, statistically significant differences were observed in the wound closure rate between the groups (control vs. Tegaderm group; control vs. PAS-2 group; Tegaderm group vs. PAS-2 group). Furthermore, when the wounds were treated for 12 days, the wound closure rate in all groups increased obviously. It was clear that there was almost completely healed in the PAS-2 group (wound close rate = 95.33%), while in the control group 11% (*p* < 0.05) of wounds were not completely healed. All the results showed that the PAS-2 group had better treatment efficacy than the Tegaderm group. This is because, compared to the Tegaderm™ film, a larger number of wettability groups (hydroxy, sulfonate, and amide) from copolymerized poly (AAm-co-SVBA) assist to trap more moisture and maintain long-term hydration micro-environments. This structural adhesive design can make the zwitterionic hydrogel matrix fit the organ wounds smoothly and perfectly, leading to the overall adsorption of excess exudates and toxins during the dynamic healing processes. Besides the features listed above, the electroactivity of PAS-2 hydrogel also contributed to promoting the wound healing process (Gharibi et al., 2015). Overall, the PAS-2 group displayed a better therapeutic effect on wound healing than commercial film dressing.

### Histological Examination

**FIGURE 7:**
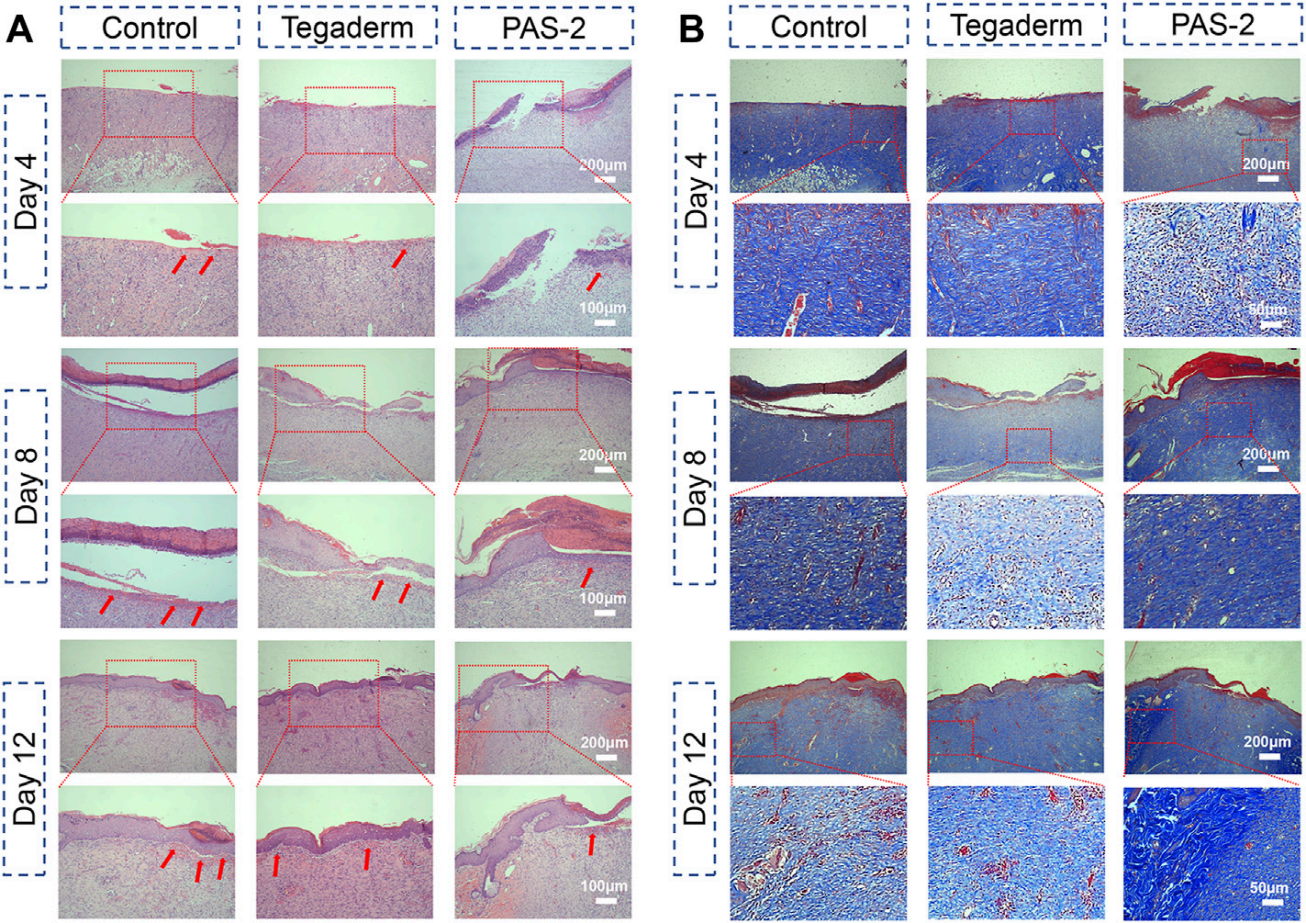
Histomorphological evaluation of the regenerated skin tissue after healing for 4, 8, and 12 days. **(A)** Images of H&E staining of the regenerated tissues in the control (gauze), Tegaderm, and PAS-2 groups after healing for 4, 8, and 12 days (red arrows: inflammatory cell). **(B)** Images of Masson’s staining of the regenerated tissues in the control (gauze), Tegaderm, and PAS-2 groups after healing for 4, 8, and 12 days.

Classical cutaneous wound healing is a complex biological process that consists of hemostasis, inflammation, re-epithelialization, innate and adaptive immune response, and tissue repair (Fang et al., 2009). To further explore the wound repair efficiency of PAS-2 hydrogel, HE and Masson’s staining was performed on the wound tissue to further observe the quality of the regenerated tissue and pathological changes in different groups. As shown in Figure 7A, the images of the PAS-2 group showed fewer inflammatory cells on the site of the wounds, which was mainly due to the scavenging effect of the polySVBA, which killed the bacteria on the wounds, but there were more inflammatory cells in the control and Tegaderm groups, which could inhibit the formation of granulation tissue and the epithelial layer. On the fourth day, there was no obvious sign of healing in the control group. In the Tegaderm group, the wound showed thickened basal membrane and sporadic keratinocytes, but the wound can be seen as the confluence of keratinocytes in the PAS-2 group. On the eighth day, the keratinocytes approaching confluence and thickened basal membrane were seen in the control and Tegaderm groups, meanwhile, we can see confluent epithelium, but nuclei not distinctly basal in the PAS-2 group. On the 12th day, the keratin and partly clear palisades can be seen in the PAS-2 group, this is a distinguishing trait of wound healing that is rarely seen in other groups. According to the scoring system of the skin wound model (Eikebrokk et al., 2019), the scores of the PAS-2 group were significantly higher than the control group (Supplementary Figure S1A). The difference was statistically significant (*p* < 0.05).

The collagen deposition in the wound was assessed by Masson staining. As can be seen from Figure 7B, the collagen in the wounds treated with hydrogel or commercial film were stained blue. Compared with the control and Tegaderm groups with less collagen, the wounds treated by the PAS-2 group could see a large amount of collagen density on day 8 and day 12. Moreover, the collagen dense of the PAS-2 group was higher than the control and Tegaderm groups (Supplementary Figure S1B) on day 12 (*p* < 0.05), indicating that the deposition of collagen could be promoted in the granulation tissue by the application of polyAAm and polySVBA.

**FIGURE 8:**
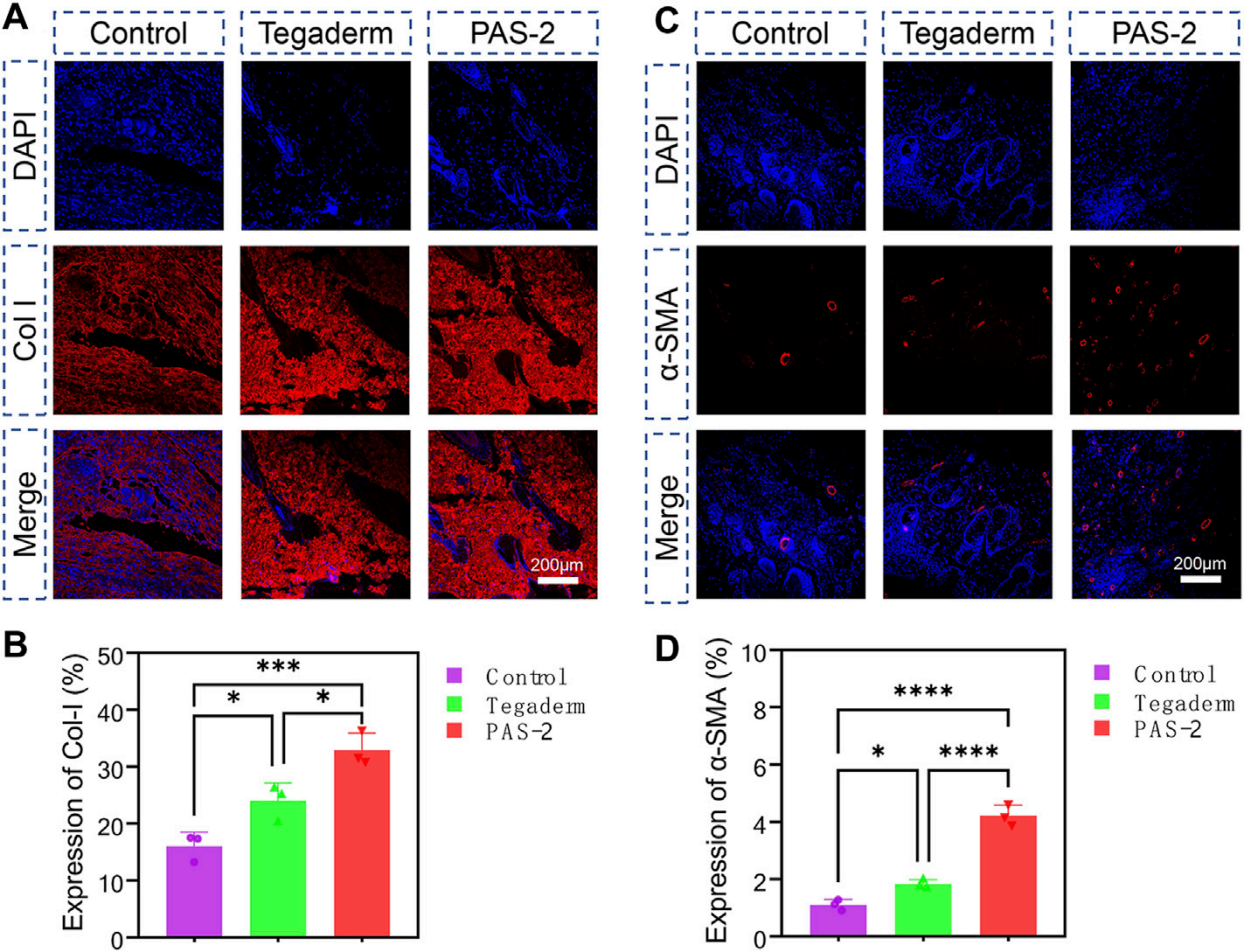
Immunofluorescence staining of Collagen I (Col I) and alpha-smooth muscle actin (α-SMA) in control, Tegaderm, and PAS-2 groups. Representative images of the regenerated wound tissue on the 12th day by immunofluorescence staining labeling with Collagen I (Col I) **(A)** and α-SMA **(C)**. Quantitative statistical analysis of the expression of Col I **(B)** and α-SMA **(D)**. *p < 0.05, **p < 0.01, ***p < 0.001, ****p < 0.0001.

The essential component for the function and structure of the skin is collagen, which includes the fibrillar collagen I and III. Collagen I is an important biomarker for the remodeling of damaged skin tissue (Cheng et al., 2020). In addition, after skin injuries, the expression of α-smooth muscle actin (α-SMA) during wound healing is important for cell migration, fibroblasts transdifferentiation, vascularization, and wound contraction (Yan et al., 2021). The expression of collagen I and α-SMA were evaluated by immunohistochemical staining of the wound tissue on day 12 to further investigate the mechanism of PAS-2 hydrogel promoting wound healing. The expression of collagen I (Figure 8A) and α-SMA (Figure 8C) in the PAS-2 group was significantly superior to the other groups, furthermore, statistical analysis confirmed that the expression of collagen I (Figure 8B) and α-SMA (Figure 8D) in the PAS-2 group was significantly higher than that in wounds treated with gauze or Tegaderm™ film (*p* < 0.05). These results showed that PAS-2 hydrogel can upregulate the expression of collagen I and α-SMA to accelerate vascular angiogenesis and promote the formation of granulation tissue. To summarize, the PAS-2 hydrogel remarkably enhanced wound healing, which was reflected as the attenuated inflammation infiltration, enhanced vascularization, collagen deposition, and remodeling.

## Conclusion

In this work, we designed and introduced a new structural zwitterion poly[3-(dimethyl(4-vinylbenzyl) ammonium) propyl sulfonate] (SVBA) into the polyAAm network to obtain a novel salt-responsive conductive hydrogel. Because of the SVBA addition, the PAS-2 hydrogel was endowed with good electroactive properties and remarkable mechanical properties. In addition, The PAS-2 hydrogel displayed excellent cytocompatibility, stable rheological property, effective antibacterial property, strong adsorption, and high penetrability. For the *in vivo* wound healing test, the PAS-2 hydrogel exhibited faster wound healing, a higher healing score, higher collagen deposition, fewer inflammatory infiltration, and more angiogenesis than the commercial film and gauze. Finally, all these results demonstrated that the salt-responsive conductive PAS-2 hydrogel film could promote the wound healing process effectively and can be a promising candidate as film wound dressing for cutaneous skin wound healing.

## Data Availability

The raw data supporting the conclusion of this article will be made available by the authors, without undue reservation.
