# Supplementary material for: Conductive Adhesive and Antibacterial Zwitterionic Hydrogel Dressing for Therapy of Full-Thickness Skin Wounds
Source: Front Bioeng Biotechnol. 2022 Feb 24;10:833887. doi: 10.3389/fbioe.2022.833887 (PMC8919325; doi:10.3389/fbioe.2022.833887)
Supplement: Supplementary file 1 [file Table1.DOCX]

Supplementary Material


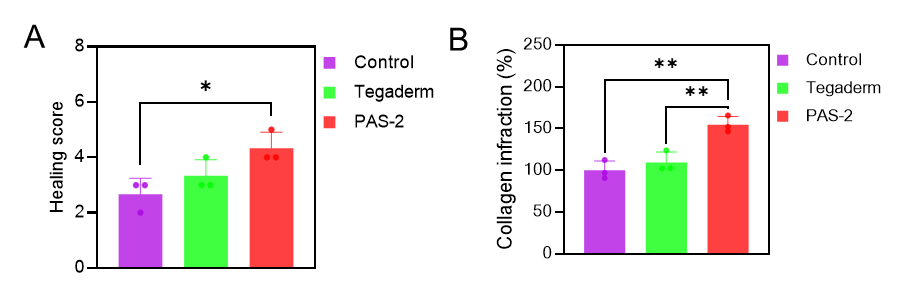
 Fig. S1. (A) Healing score in ex vivo skin wound model after treated with gauze (control), Tegaderm film and poly (AAm-*co*-SVBA)-2 (PAS-2) hydrogel on day 12. (B) Relative coverage of collagen in the regenerated wound tissue after healing for 12 days. **P*＜0.05, ***P*＜0.01, ****P*＜0.001, *****P*＜0.0001.
